# Halogen bonding and hydrogen bonding fluorescent anion sensing at the solid–liquid interface

**DOI:** 10.1039/d5sc09194b

**Published:** 2026-01-05

**Authors:** Robert Hein, Mohamed Sharafeldin, Edward J. Mitchell, Jason J. Davis, Paul D. Beer

**Affiliations:** a Department of Chemistry, Chemistry Research Laboratory, University of Oxford South Parks Road Oxford OX1 3QZ UK paul.beer@chem.ox.ac.uk; b Organic Chemistry Institute, University of Münster Corrensstraße 40 48149 Münster Germany robert.hein@uni-muenster.de

## Abstract

Halogen bonding (XB) has emerged as a powerful non-covalent interaction in anion supramolecular chemistry and is now well-established for the recognition and sensing of various environmentally and biologically relevant anionic species in solution. To translate the significant potential of XB-mediated anion binding to real-world sensor application requires both a consideration of XB material device integration and utilisation in water; key areas that remain noticeably underdeveloped. Addressing this challenge, we herein report the first example of a XB monolayer architecture for the detection of anions at the solid–liquid interface *via* fluorescence, enabling sensor re-use and detection of various anions in both organic solvent and in pure water. To this end, a BODIPY-bis(iodo)triazole receptor was covalently immobilized onto glass slides *via* amide bond formation. Detailed unprecedented comparisons of the anion binding and sensing performance of this XB interface with both an analogous solution-phase XB receptor as well as their hydrogen bonding (HB) congeners revealed that the system's fluorescence sensing performance is largely retained upon surface immobilization for all tested anions. Interestingly, and in contrast to solution-phase experiments, the XB interface outperformed the HB interface in all cases, both in terms of anion binding strength and signal response. These observations pave the way for a rational translation of established solution-phase fluorescent XB anion receptors to molecular film sensing formats, which, as shown here, both support sensor re-use and circumvent solubility constraints.

## Abstract 2

Glass substrates, functionalised with BODIPY-(iodo)triazole host monolayers, facilitate anion detection *via* fluorescence emission changes in both organic solution and pure water circumventing solubility constraints and enabling sensor reuse.

## Graphical abstract

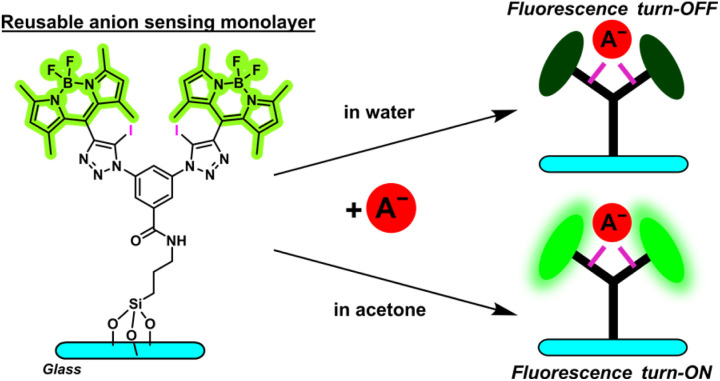

## Introduction

Stimulated by their importance in a wide variety of technological, environmental and biological settings, the sensing of anions continues to be of intense interest and remains a formidable challenge.^1,2^ As a result, enormous attention has been directed at the development of increasingly sensitive and selective sensors, most notably those based on synthetic supramolecular anion receptors containing optical or electrochemical signaling units.^3–7^ The principle of this host–guest sensing approach is academically well-established but is notably underused in real-life applications. It remains largely restricted to solution-phase sensing studies; a setting in which its most notable advantage over reaction-based sensory probes (chemodosimeters), the reversibility of the non-covalent host–guest interaction, cannot be easily exploited. Only upon integration of such sensors into suitable materials can the sensor be re-used while concomitantly providing a clear path towards device integration and, for example, continuous, real-time flow ion sensing.^8–10^ A particularly appealing format is presented by 2-dimensional monolayer architectures on solid supports; in general terms they represent an archetypical, simple and highly (chemically) tunable platform for the construction of interfacial ion sensors. They not only utilize minimal amounts of sensory probe and circumvent solubility constraints, but also respond much more rapidly to changes in analyte concentration than (polymeric) 3D-materials such as membranes (wherein diffusion may be a limiting factor) and are thus ideally suited for continuous, real-time sensing applications.^11,12^

As a result, a range of monolayer architectures based on synthetic ion receptors, in particular self-assembled monolayers (SAMs) on gold or glass, have been fabricated for both optical and electrochemical ion sensing (cation and anion).^13–23^ However, this remains significantly underdeveloped, especially in the case of anions, which can largely be attributed to their larger size, lower charge-density and stronger hydration in comparison to isoelectronic cations, such that their supramolecular recognition is more challenging.^1^

To address these challenges, significant attention has been directed at the exploitation of sigma-hole interactions, in particular halogen bonding (XB)^24–26^ as a particularly powerful non-covalent interaction for the recognition and sensing of anions, typically displaying significantly enhanced anion binding strength and selectivity over hydrogen bonding (HB).^27–29^ This has enabled sensitive anion sensing in numerous solution-phase formats, including in pure water.^30,31^ We, and others, have recently also demonstrated that XB-based anion receptors can be leveraged for the construction of potent SAM-based electrochemical anion sensors, where, depending on the specific sensing format, significant alterations in sensitivity or selectivity in comparison to the analogous solution-phase sensors were observed.^32–38^ In contrast, the exploration of XB-mediated optical anion sensing in material formats is notably underdeveloped^39^ and is, to the best of our knowledge, entirely unprecedented for the fluorescence sensing of anions at monolayers.

Herein, we demonstrate that immobilization of XB boron-dipyrromethene (BODIPY)-containing fluorescent anion receptors onto transparent glass substrates facilitates sensitive sensing of biologically and environmentally relevant anions in both organic media and, importantly, pure water with improved performance over analogous HB based sensors. The receptive BODIPY motif is not sufficiently water soluble (even when PEGylated) but can be immobilized to support effective purely aqueous phase sensing and facile sensor re-use.

## Results and discussion

### Design and synthesis of fluorescent BODIPY receptors

**Scheme 1:**
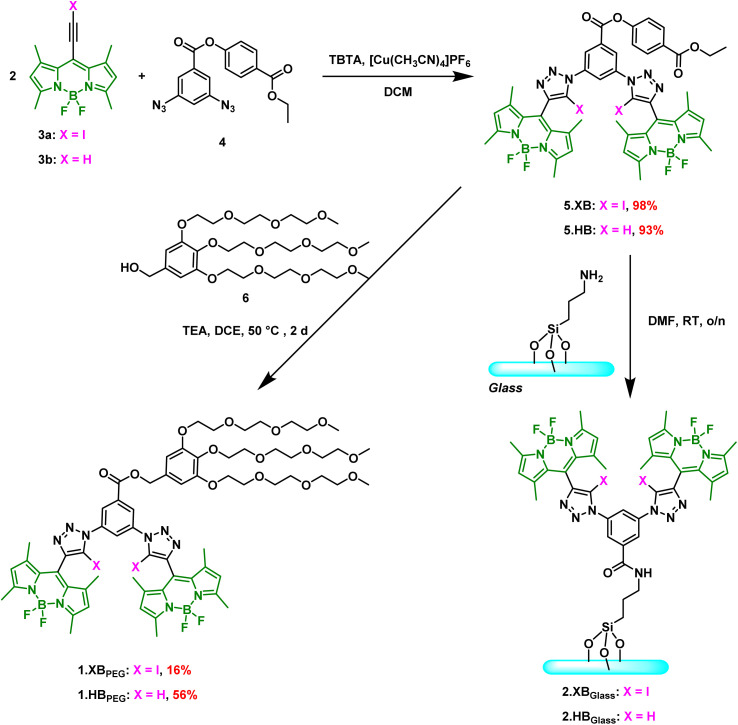
Synthetic routes towards 1.XB/HB_PEG_ and 2.XB/HB_Glass_.

The XB and HB bis(BODIPY-(iodo)triazole) receptors were designed to contain the well-established, potent bis(iodo)triazole anion binding motif,^28,40^ onto which fluorescent BODIPY reporter groups were directly appended (Scheme 1). We have recently demonstrated that this core receptor scaffold is a versatile and sensitive solution-phase fluorescence sensor for various anions in acetone with significant fluorescence turn-ON, especially in the presence of chloride and bromide even at µM levels.^41^

To enable further functionalisation of this sensor system, specifically surface-immobilisation as well as the installation of solubilising groups, we designed a novel variant containing an ethyl-paraben-derived active ester 5.XB/HB. Synthesis of this precursor was achieved in high yields of >93% by copper(i)-catalysed azide alkyne cycloaddition (CuAAC) between BODIPY iodo/proto alkyne 3a/b ^41,42^ and bis-azide 4.^31^ Transesterification of 5.XB/HB with tris(triethylene glycol)benzyl alcohol 6 ^43^ in 1,2-dichloroethane (DCE) then afforded the PEG-appended 1.XB/HB_PEG_ in moderate to modest yields (16–56%), which were prepared to enable comparative solution-phase sensing studies in a range of solvent systems, including predominantly aqueous media. Alternatively, amidation of 5.XB/HB with amine-containing glass surfaces enabled generation of 2.XB/HB_Glass_ (*vide infra*). For further experimental details and full compound characterisation by NMR and high-resolution MS see SI Section S2.

### Solution-phase fluorescent anion sensing

**Fig. 1:**
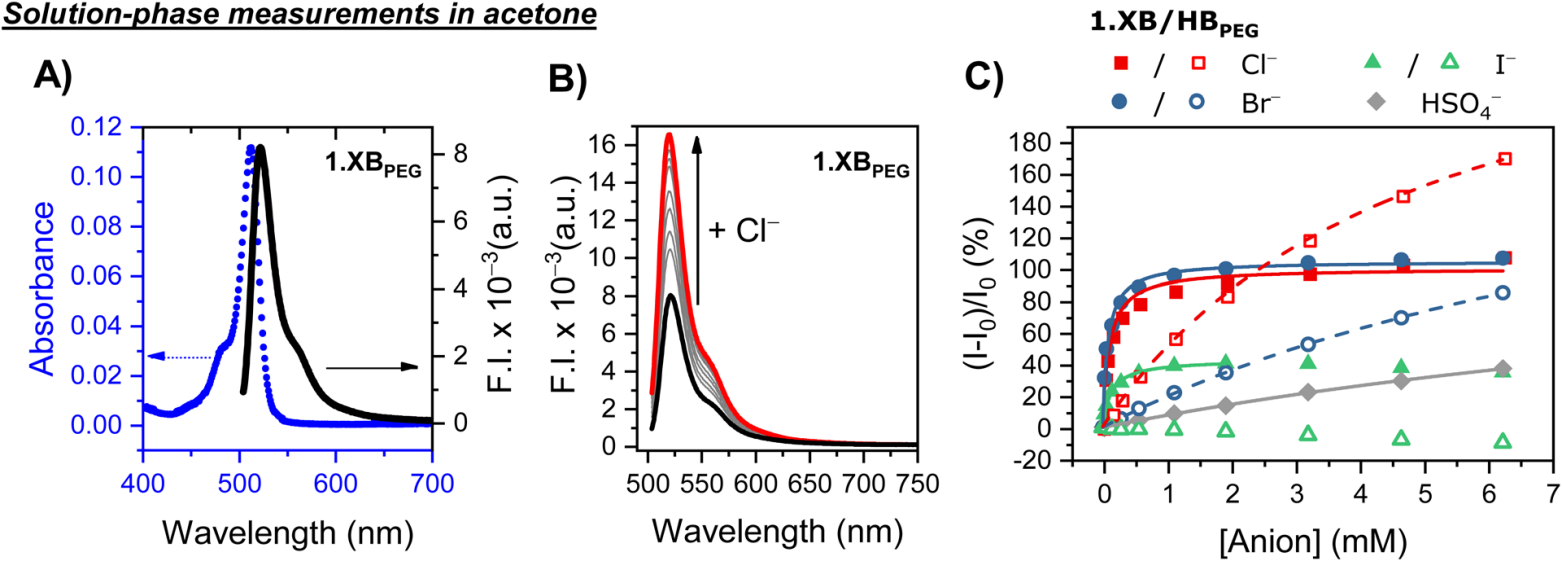
Photophysical properties of 1 µM 1.XB/HB_PEG_ in acetone. (A) Absorbance (dashed blue line) and fluorescence emission intensity (solid black line) of 1.XB_PEG_. (B) Fluorescence response of 1.XB_PEG_ upon addition of increasing amounts of Cl^−^. (C) Relative fluorescence emission response of 1.XB_PEG_ (filled symbols) and 1.HB_PEG_ (empty symbols) at *λ*_max_ upon addition of various anions. The solid/dashed lines correspond to fits according to a 1 : 1 host–guest stoichiometric binding model.

Only minor differences in absorbance and emission wavelengths between both receptors 1.XB/HB_PEG_ and between solvents were observed in all cases (acetone, DMF, CH_3_CN, CH_3_CN/H_2_O 3 : 7); see Table 1 for a collation of the most relevant photophysical properties. Specifically, the novel receptors displayed a well-defined BODIPY-based absorbance (*λ*_max, abs_ ≈ 513 nm) and fluorescence emission (*λ*_max, em_ ≈ 526 nm) with a small Stokes shift, as representatively shown for 1.XB_PEG_ in acetone in Fig. 1A.

**Table 1:** Photophysical properties of 1.XB/HB_PEG_ and 2.XB/HB_Glass_ receptors in acetone and water

	Acetone				Water			
	1.XB/HB_PEG_		2.XB/HB_Glass_		1.XB/HB_PEG_ a		2.XB/HB_Glass_	
	XB	HB	XB	HB	XB	HB	XB	HB
*λ*_max, abs_ (nm)	512	509	n.a.	n.a.	512	510	n.a.	n.a.
*ε* (M^−1^cm^−1^)	112 000	195 000	n.a.	n.a.	145 000	180 000	n.a.	n.a.
*λ*_max, em_ (nm)	522	520	524	523	526	523	528	528
Stokes shift (nm)	10	11	n.a.	n.a.	14	13	n.a.	n.a.

a For solubility reasons 30% CH_3_CN was added for solution-phase anion binding studies of 1.XB/HB_PEG_. n.a. – not available.

The solution-phase anion binding and sensing capabilities of these fluorescent receptors were initially investigated in acetone. As shown in Fig. 1B and C, addition of the halide anions Cl^−^, Br^−^ or I^−^ as their tetrabutylammonium (TBA) salts induced significant fluorescence turn-ON (up to 100% relative emission intensity increase, Table S2) for 1.XB_PEG_ with strong binding of up to *K* = 12 700 M^−1^ for bromide and slightly weaker binding for chloride (*K* = 9980 M^−1^) and iodide (*K* = 8050 M^−1^), as deduced from fitting of the binding isotherms to a 1 : 1 host–guest stoichiometric binding model (Table 2). These observations are in good agreement with previous studies on related 1,3-bis(iodo)triazole benzene anion receptors, which also display strong 1 : 1 host–guest stoichiometric anion binding through convergent XB/HB interactions, as deduced by ^1^H NMR or optical anion binding titrations as well as computational studies.^40,41,44,45^

**Table 2:** Anion binding constants *K* (M^−1^)^a^ of 1.XB/HB_PEG_ and 2.XB/HB_Glass_ in acetone and water obtained by global fitting of the fluorescence binding isotherms to a 1 : 1 host–guest stoichiometric binding model (solution) or Langmuir isotherm (interface)

	Acetone				Water			
	1.XB_PEG_	1.HB_PEG_	2.XB_Glass_	2.HB_Glass_	1.XB_PEG_^b^	1.HB_PEG_^b^	2.XB_Glass_	2.HB_Glass_
Cl^−^	9980 ± 700	200 ± 3	1590 ± 60	1090 ± 60	—	—	—	—
Br^−^	12 700 ± 460	90 ± 2	1510 ± 60	97 ± 72	—	—	—	—
I^−^	8050 ± 120	—	—	—	63 ± 1	10 ± 1	74 ± 2	47 ± 1
SCN^−^	—	—	—	—	29 ± 1	12 ± 1	55 ± 2	27 ± 1

a All errors represent mathematical fitting errors obtained by global fitting of the isotherms at multiple different wavelengths.

b For solubility reasons 30% CH_3_CN was added for solution-phase anion binding studies.

The strong enhancement of fluorescence emission can be attributed to receptor rigidification by anion binding and the associated suppression of non-radiative decay pathways. Specifically, rotation around the *meso*-BODIPY bond is restricted when an anion binds to the receptor, thereby conformationally “locking” the BODIPY reporter groups which decreases the rate of internal conversion and thereby enhances fluorescence emission.^41,46^ While Br^−^ and Cl^−^ induced a near identical emission increase, the response of 1.XB_PEG_ in the presence of I^−^ was notably smaller and even slightly decreased at higher anion concentrations. These observations can most likely be attributed to additional non-specific quenching by this anion, *e.g.* through heavy atom effects,^47^ which counteract the above-described rigidification effect and thus lead to an overall smaller response magnitude and at higher concentrations even to a non-monotonic (slightly diminished) response.^41^

The bisulfate anion induced much smaller fluorescence perturbations, with weak binding to 1.XB_PEG_ (*K* = 60 M^−1^). Thus, no further experiments were carried out with this anion.

In comparison, halide binding strength at 1.HB_PEG_ was attenuated by almost two orders of magnitude for chloride and bromide, with significant fluorescence turn-on (up to 170% for Cl^−^), only at higher concentrations (>1 mM). Moreover, iodide induced only very weak fluorescence turn-OFF, presumably because binding (and associated receptor rigidification) is negligible and non-specific quenching dominates. Overall, these observations are in excellent agreement with previous studies of the core sensor scaffold without the additional ester functionality (Fig. S14), for which the same response trends were observed.^41^ However, due to its electron-withdrawing nature, the additional active ester substituent not only enables further functionalization, as demonstrated here for the appendage of PEG-groups or surface immobilization, but also further polarizes the (iodo)triazole XB or HB bond donors, thereby enhancing anion binding strength. For example, for 1.XB_PEG_ bromide binding strength is enhanced by ∼50%, while the relative increase was even larger for binding of both Br^−^ and Cl^−^ to 1.HB_PEG_ (up to ∼3× increase relative to the non-functionalized analogue, however at much lower absolute binding strength in comparison to the XB congener), see Table S1 for detailed comparisons.

Having established the general anion binding and fluorescent sensing potency of 1.XB/HB_PEG_ in organic solvent, attention focused on investigating their capabilities in water. While soluble at 1 µM in water, the fluorescence intensity of both receptors were quenched around 100-fold in comparison to organic solvents, indicative of aggregation, thus preventing detailed anion binding/sensing studies. Only addition of detergent or 30% CH_3_CN as co-solvent induced disaggregation and fluorescence recovery (see Fig. S11 and 12). Fluorescent anion sensing studies were then undertaken in this predominantly aqueous solvent system of H_2_O/CH_3_CN 7 : 3 with a range of anions, including the halides as well as the weakly hydrated anions SCN^−^ and ClO_4_^−^. As shown in Fig. S13, only iodide and thiocyanate induced significant responses, as characterised by fluorescence emission quenching, which for both 1.XB_PEG_ and 1.HB_PEG_ was larger for I^−^ than SCN^−^, with >50% quenching but only at high anion concentrations. This emission quenching again most likely arises from heavy-atom effects. In good agreement with the shallow response isotherms are the much lower fitted anion binding constants for both receptors in this aqueous medium, whereby 1.XB_PEG_ (*K*_I^−^_ = 63 M^−1^ and *K*_SCN^−^_ = 29 M^−1^) is still a superior host, both in terms of binding strength and response magnitude, than its HB counterpart 1.HB_PEG_ (*K*_I^−^_ = 10 M^−1^ and *K*_SCN^−^_ = 12 M^−1^), again underscoring the potential of XB interactions in anion recognition and sensing.^24^ The fact that only SCN^−^ and I^−^ induced notable responses can most likely be attributed to their lower enthalpy of hydration. As briefly discussed above, for I^−^ the emission quenching is probably related to heavy-atom effects, while quenching by SCN^−^ potentially arises from photo-induced electron transfer (PET) of this electron-rich anion, as previously reported in other fluorescent ion sensors.^48,49^ Binding of these anions in water presumably still induces receptor rigidification (and associated emission enhancement as observed in acetone), but due to weaker anion binding and the resulting need for higher anion concentrations, PET/heavy atom quenching pathways overall dominate thereby leading to emission turn-OFF.

### Interfacial fluorescent anion sensing

Immobilisation of the fluorescent receptors was achieved by overnight immersion of commercially available amine-coated glass substrates into a 0.1 mM solution of the active-ester 5.XB/HB in anhydrous DMF, affording 2.XB/HB_Glass_ (Scheme 1). Successful receptor coupling *via* amide-bond formation was confirmed by fluorescence measurements (*vide infra*), as well as contact angle measurements. The unfunctionalised, native aminated glass displayed a water contact angle of 40.1 ± 1.6° indicative of a relatively hydrophilic interface. Upon immobilisation of the more hydrophobic receptors small, but reproducible, increases in water contact angle to 45.7 ± 0.8° and 42.8 ± 1.6° of 2.XB_Glass_ and 2.HB_Glass_ were observed, respectively. These small changes are indicative of a relatively sparse receptor surface coverage, while the slightly larger water contact angle for the XB interface is likely reflective of the higher hydrophobicity of the iodotriazole motif.^33,34^

**Fig. 2:**
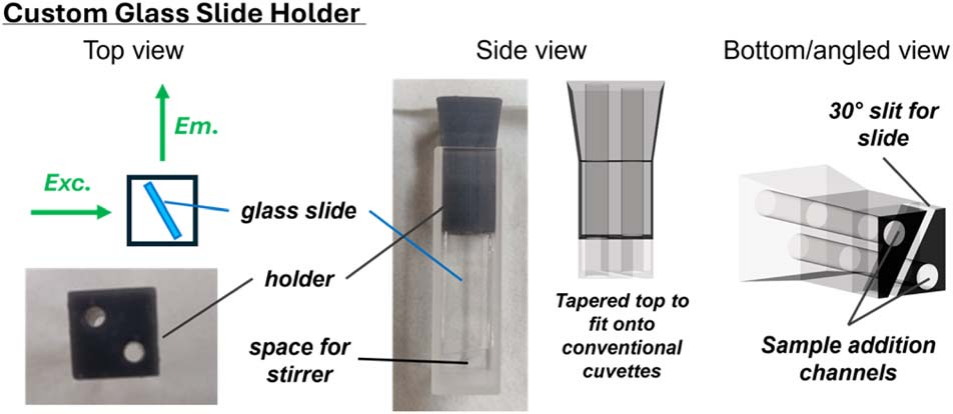
Schematic depictions and photos of custom-made 3D-printed cuvette holder (grey) which enabled fluorescence measurements of the functionalized glass slides with a fixed geometry (here 30° through the slide) and addition of anion aliquots. For the actual fluorescence measurements, the cuvette was additionally filled with a desired solvent (acetone or water) and equipped with a small stir bar at the bottom of the cuvette.

While the sub-monolayer receptor coverage precluded accurate UV/vis measurements, the high brightness of the BODIPY fluorophore generated a sufficiently strong emission for detailed sensing studies. To enable fluorescence measurements in a standard fluorescence spectrometer with a fixed 90° measurement geometry, we designed custom-made 3D-printed holders which allowed us to position the functionalized glass slides in a well-defined and fixed geometry within standard 1 × 1 cm fluorescence cuvettes (Fig. 2). Importantly, the holder was designed in a manner which allowed addition of aliquots of anion solution to the cuvette such that the concentration could be systematically varied/titrated, without altering the position of the glass slide, thereby enabling detailed binding and sensing studies. To this end, the custom holder was designed such that the glass slide was suspended from the top of the cuvette bringing the functional surface into the light beam, but with sufficient space at the bottom for a miniature stirring bar (Fig. 2). Through holes in the holder, titrant solution could then be added *via* Hamilton syringes into a continuously stirred solution. The angle of the slide was optimized to minimize interference from scattered excitation light. Specifically, we found a 30° angle with respect to the emission light path and a geometry where the detector was on the back-side of the glass slide (*i.e.* a light “through” the slide geometry) to be optimal in our case; see Section SI4 for further details on the holder design.

**Fig. 3:**
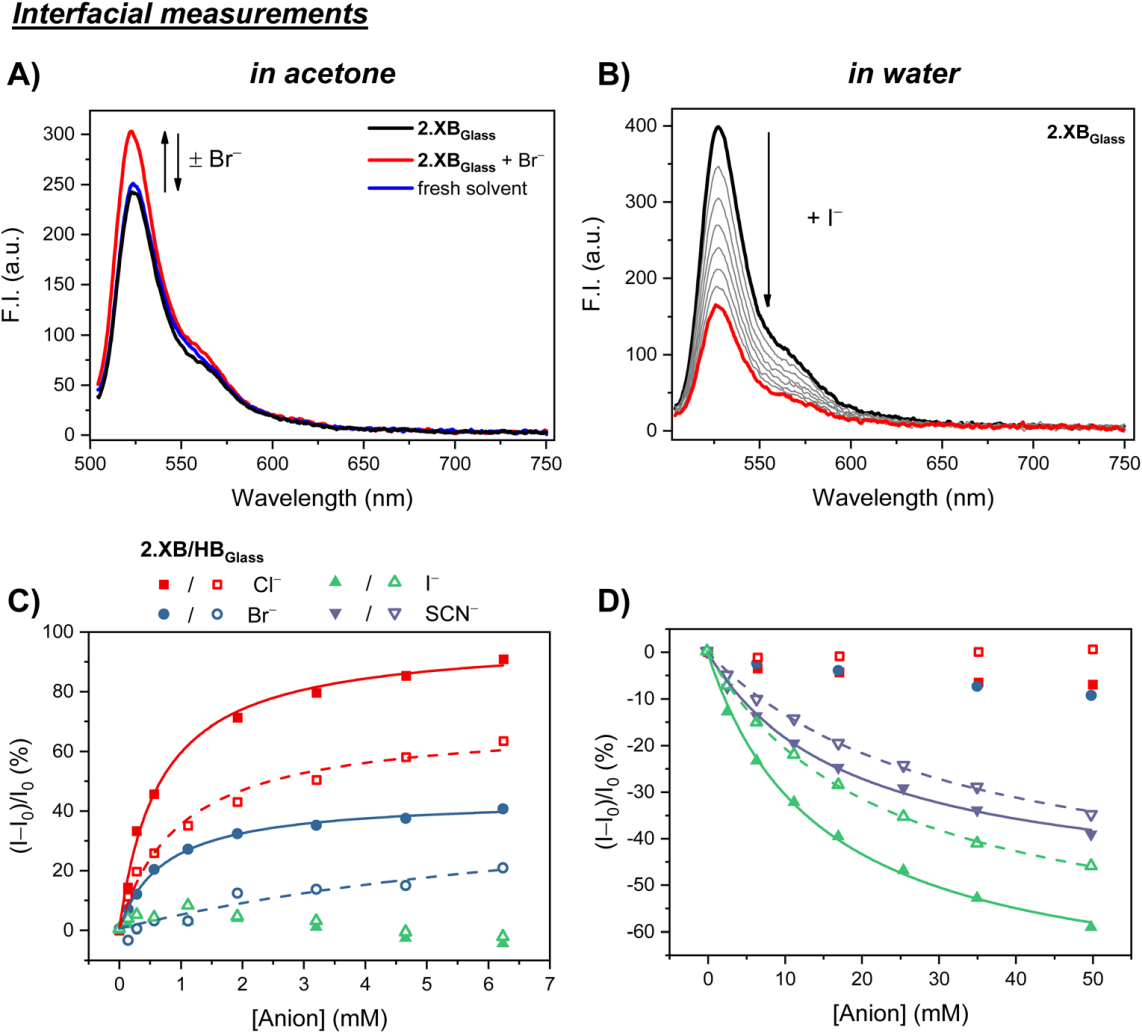
Photophysical properties of 2.XB/HB_Glass_ in acetone (A and C) or water (B and D). (A) Fluorescence spectra of 2.XB_Glass_ in acetone (black), upon addition of Br^−^ (red) and subsequent solvent exchange back to pure acetone (blue), highlighting the reversibility of the sensory interface. (B) Fluorescence response of 2.XB_Glass_ in water upon addition of increasing amounts of I^−^. (C) and (D) Relative fluorescence emission response of 2.XB_Glass_ (filled symbols) and 2.HB_Glass_ (empty symbols) upon addition of various anions at *λ*_max_ in acetone (C) and water (D). The solid/dashed lines correspond to fits according to a 1 : 1 stoichiometric host–guest binding Langmuir model.

As shown in Fig. 3A and B, the fluorescence of 2.XB/HB_Glass_ was, due to a much smaller number of molecules probed, expectedly strongly attenuated in comparison to the above-discussed solution-phase studies,††As a result of the low fluorescence intensity, the emission of the glass slides is not visible by the naked eye but can be observed by fluorescence microscopy (Fig. S15). but the overall shape of the emission was virtually unchanged with a very similar *λ*_max_ both in acetone and in water. Upon repeat measurements, no change of the glass slide emission of was observed, confirming the stability of the interface and lack of photobleaching. Importantly, control experiments confirmed specific, covalent recruitment of the receptors onto the glass surface. Specifically, non-aminated, bare glass slides showed negligible fluorescence after exposure to the 5.XB/HB active esters under otherwise identical immobilisation conditions. Additionally, even after extensive ultrasonication of 2.XB/HB_Glass_ in a concentrated aqueous solution of the detergent Triton X-100 no significant change in fluorescence intensity was observed, confirming that the receptors are not simply physisorbed. In contrast, after brief exposure of the receptive surfaces to fresh piranha solution (H_2_SO_4_/H_2_O_2_ 3 : 1 v/v) the fluorescence was almost fully erased, as the receptive film was destroyed.

Detailed investigations of the anion sensing properties of 2.XB/HB_Glass_ were then carried out with the same anions as detailed for solution-phase sensing studies (*vide supra*). In acetone, only chloride and bromide induced a significant fluorescence turn-ON for 2.XB/HB_Glass_, as representatively shown for 2.XB_Glass_ in the presence of bromide in Fig. 3A, while iodide induced no notable response. Crucially, upon washing the sensor surface with solvent and subsequent measurements in pure acetone the initial fluorescence intensity of 2.XB_Glass_ was recovered (Fig. 3A). This highlights that the sensor can be easily reused or, in principle, even be employed for continuous, real-time flow sensing.

Systematic binding/sensing titrations were then carried out for various anions across a wide concentration range for the interfacial sensors in both acetone and also water. Importantly, the sensing performance of the probes are qualitatively largely retained upon surface immobilization at least for the set of anions tested herein; in acetone fluorescence turn-ON for Cl^−^ and Br^−^ was observed, while these more hydrophilic halides induced no noticeable response in water. In the aqueous medium only I^−^ and SCN^−^ induced responses, as characterised by significant emission turn-OFF, in agreement with solution-phase studies of 1.XB/HB_PEG_ in H_2_O/CH_3_CN 7 : 3.

Nevertheless, subtle differences between the interfacial and solution-phase sensing properties were observed, in particular in acetone. In acetone solution all tested halides induced notable emission changes of 1.XB_PEG_, while the analogous surface sensor 2.XB_Glass_ did not respond to iodide, and thus has a higher selectivity. In addition, the overall response magnitudes of the surface sensors in acetone are somewhat lower for all anions, in particular for Br^−^ and I^−^. As a result, the maximum response of 2.XB_Glass_ towards Cl^−^ and Br^−^ differed substantially, with maximum relative response intensities *I*_max_ of +98% and +44%, respectively, while in solution near identical maximum responses of ∼+100% were observed for these two anions (see Table S2). This effect is even more drastic for the HB sensing systems; in solution 1.HB_PEG_ showed very large emission turn-ON of up to +306% and +238% for Cl^−^ and Br^−^, respectively, while 2.HB_Glass_ displayed attenuated *I*_max_ of +70% and ∼+50%. This difference in overall maximum response magnitude between the solution-phase and interfacial settings potentially arises from restricted conformational flexibility at the surface. Interestingly, this effect is apparently less pronounced in water, where for I^−^ sensing virtually no difference in *I*_max_ was observed between 1.XB/HB_PEG_ and 2.XB/HB_Glass_. Only for SCN^−^ a somewhat lower maximum emission turn-OFF was observed for the interfacial sensing format.

To further quantify and compare the interfacial sensor performance, anion binding constants of 2.XB/HB_Glass_ were obtained by fitting of the fluorescence binding isotherms to the well-established Langmuir adsorption model: 
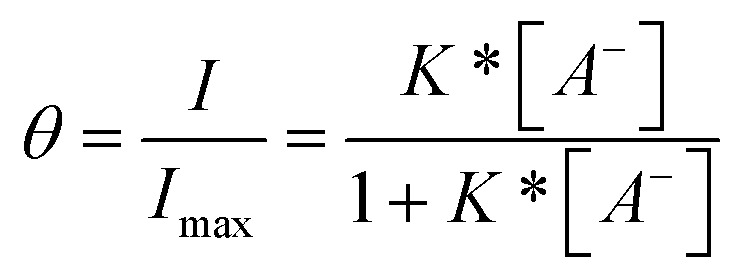
 where *θ* is the fractional surface coverage, which is here read-out as fractional fluorescence intensity *I*/*I*_max_. In all cases good fits were observed (*R*^2^ > 0.915), suggesting that the receptors on the interface are not interacting, are all in the same environment and that the host–guest binding stoichiometry is also 1 : 1.

As shown in Table 2 the fitted interfacial binding constants are generally in a similar range as those observed in solution, especially in water. In acetone, anion binding to the interface was somewhat attenuated in almost all cases, with the exception of chloride binding to 2.HB_Glass_, which was slightly stronger than in solution. Nevertheless, in all cases 2.XB_Glass_ outcompetes its HB congener, both in terms of binding strength and response magnitude.

Overall, these observations confirm that the binding and sensing performance and general selectivity trends of both receptors are largely retained upon surface-immobilisation, at least for this receptor system and at comparatively low surface coverage. In this context, it should be noted that the low receptor surface coverage is not problematic, as long as a sufficiently high fluorescence signal is obtained (as is the case here). In fact, a very dense receptor decoration on the glass surface may be detrimental due to fluorescence emission quenching/alteration arising from intermolecular fluorophore interactions (akin to the aggregation-induced quenching observed for 1.XB/HB_PEG_ in aqueous solution (Fig. S11 and 12)).

While enhanced anion sensing/binding performance is typically observed for interfacial electrochemical voltammetric anion sensors,^34,36^ and has also been reported in select cases for nanoparticle-based fluorescent anion sensors,^50^ this often comes at the expense of altered and/or lowered selectivity. In contrast, the platform reported herein notably enables predictive translation of solution-phase XB sensing performance that is tuneable by synthetic design, to a reuseable interfacial configuration which facilitates device integration and circumvents solubility constraints.

## Conclusions

The development of an improved and versatile sensing scaffold containing a bis(iodo)triazole XB or HB binding site, BODIPY fluorescent reporter groups as well as an active-ester handle for further functionalization, enabled the construction of four new sensors for the detection of anions in organic and aqueous media. This includes the first example of a XB interfacial fluorescent anion sensor, capable of repeat sensor use and detection of various anions in acetone as well as SCN^−^ and I^−^ in pure water. Detailed comparisons of the anion binding and sensing performance between both XB/HB and solution-phase/monolayer-based architectures revealed that probe surface confinement occurs with retention of their sensing properties. The XB sensors performed particularly well in all scenarios, while the HB system, which in all cases displayed weaker anion binding, also suffered very significant drop-off in response magnitude upon surface-confinement in organic solvent. Significantly, the interfacial sensors enabled anion sensing in pure water, where even the highly optimized, PEG-containing 1.XB/HB_PEG_ were not sufficiently soluble, thereby paving the way for the further rational exploration of XB in real-life relevant sensing devices. While demonstrated here for the sensing of anions in solution, we expect this or similar interfaces to also be capable of sensing other neutral Lewis bases^51^ or even gases.^52,53^

## Author contributions

R. H. carried out all experimental work and analysis with the help of E. J. M. (synthesis) and M. S. (3D printing). R. H. and P. D. B. conceived the project and wrote the original paper draft which was edited by R. H., M. S., J. J. D. and P. D. B. R. H., J. J. D. and P. D. B. supervised the project. P. D. B. acquired funding.

## Conflicts of interest

There are no conflicts to declare.

## Supplementary Material

SC-OLF-D5SC09194B-s001

## Data Availability

The data supporting this article have been included as part of the supplementary information (SI). Supplementary information: experimental details and further data. See DOI: https://doi.org/10.1039/d5sc09194b.
