# Supplementary material for: Halogen bonding and hydrogen bonding fluorescent anion sensing at the solid–liquid interface
Source: Chem Sci. 2026 Jan 5;17(8):4034–41. doi: 10.1039/d5sc09194b (PMC12810957; doi:10.1039/d5sc09194b)
Supplement: SC-017-D5SC09194B-s001 [file SC-017-D5SC09194B-s001.pdf]

Supplementary Information (SI) for Chemical Science.
This journal is © The Royal Society of Chemistry 2026

Supporting Information

Halogen Bonding and Hydrogen Bonding Mediated Fluorescent Anion Sensing at the Solid-Liquid Interface

Robert Hein[1,2,*], Mohamed Sharafeldin[1], Edward J. Mitchell[1], Jason J. Davis[1] and Paul D. Beer[1,*]

1) Department of Chemistry, University of Oxford, South Parks Road, Oxford OX1 3QZ, U.K.

2) Organic Chemistry Institute, University of Münster, Corrensstraße 40, 48149 Münster, Germany

*robert.hein@uni-muenster.de
*paul.beer@chem.ox.ac.uk

Contents

1. Instrumentation and General Experimental Details 2
2. Synthesis 4
3. Surface-Functionalisation 13
4. 3D-printed Holder for Optical Measurements 13
5. Optical Measurements 16
6. References 18

1. Instrumentation and General Experimental Details

General Information

All commercially available chemicals and solvents were used as received without further purification. Dry solvents were degassed with N_2 and dried on a Mbraun MPSP-800 column. Ultrapure water was obtained from a Milli-Q system (18.2 MΩcm). Mass spectrometry was performed on a Bruker micrOTOF. NMR spectra were recorded on Bruker NMR spectrometers (AVIII HD 600 or AVIII HD 500 or AVIII HD 400). Chromatography was performed using silica gel (particle size: 40-63 µm).

Optical Measurements

UV-vis and fluorescence measurements were carried out on a Duetta spectrofluorometer (Horiba) using quartz cuvettes with a path length of 4 or 10 mm. Spectroscopic grade acetone and CH_3CN (both from Alfa Aesar) were used throughout. For measurements in aqueous media, Milli-Q water (18.2 MΩcm) was used. All solutions were passed through syringe filters prior to the experiments. In all cases at least three repeat fluorescence spectra were recorded to ensure signal stability.

<u>Solution-phase fluorescence experiments:</u>

All experiments were carried out using a concentration of 1 µM of the receptors in 4 mm pathlength quartz cuvettes. For anion titration studies a 1 mL solution of the receptor was titrated with aliquots of a concentrated solution of TBA-anion in the same receptor solution to ensure a constant receptor concentration. Unless otherwise noted, all fluorescence spectra were acquired with a wavelength of excitation of 485 nm, 5 nm excitation and emission slits.

<u>Interfacial fluorescence experiments:</u>

Immediately before experiments the functionalised glass slides were thoroughly dried under a stream of nitrogen and then mounted in the 3D-printed holder. The holder with the slide was then pushed into an open 1 x 1 cm cuvette containing the blank solvent (acetone or water) and a small stir bar, until the slide and holder were securely fixed and didn´t move. Most typically, 1500 µL of initial filling solution (acetone or water) were added to the cuvette, ensuring that there were no air bubbles stuck to the cuvette or slide. This volume was chosen such that the light beam of the spectrometer was fully within the solution, but that there was enough free volume for the addition of the anion titrant aliquots, which were added as concentrated solution as the sodium or TBA salts of the anions in the same solvent (typically up to 500 µL total added volume). The aliquots were carefully added via Hamilton syringe through the holes in the cuvette into a continuously stirred solution without touching the

holder or cuvette to prevent changing the position of the glass slide. The total volume at the end was chosen such that the solution did not touch the holder. Each titration was carried out on a separate/new slide. An excitation wavelength of 485 nm as well as emission and excitation slit widths of 10 nm were used.

Data analysis

All data analysis and fitting was carried out with OriginPro. To obtain solution-phase binding constants, the obtained fluorescence binding isotherms were fitted accorded to the established 1:1 host-guest stoichiometric binding model.[1] Interfacial binding constants were obtained by fitting to a standard Langmuir adsorption isotherm. All binding constants were obtained by using a global fitting procedure of at least 5 different wavelengths, typically in between 520 – 560 nm.

For interfacial fluorescence measurements the spectra were smoothed via 5-point adjacent averaging.

Contact Angle Measurements

Static water contact-angle measurements were performed on a FTA1000B goniometer (First Ten Ångstroms) equipped with a manually operated microliter syringe. A sessile drop of deionised water (18.2 MΩ cm) was used as the wetting liquid. Data analysis was carried out with the FTA32 software and all droplets were fitted to a spherical model.

2. Synthesis

BODIPY alkynes **3a**[2] and **3b**[3] and tris(triethylene glycol)benzyl alcohol **6**[4] were prepared according to literature procedures.

Compound **4** was prepared according to improved literature procedures[5-7] as detailed in the following (Scheme S1).

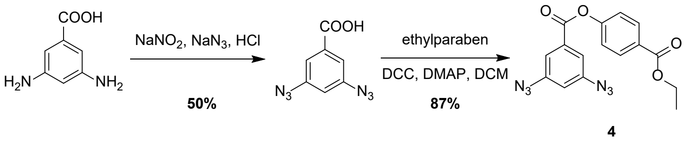

Scheme S1. Synthetic route towards **4**.

<u>3,5-diazidobenzoic acid</u>

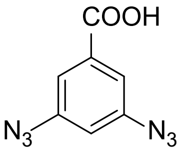

Synthesised according to adapted literature procedures:[5-6] 5 g of 3,5-diaminobenzoic acid (33 mmol, 1 equiv.) was dissolved in 40 mL 6 M HCl. 6.7 g sodium azide (100 mmol, 3.3 equiv.) was carefully dissolved in this solution, which was then cooled to 0 °C. 10 g of sodium nitrite (140 mmol, 4.25 equiv.) were added portion-wise over 2 h. The reaction was then stirred at room temperature overnight. The product was then extracted with EtOAc (3x), the organic phase washed with 1 M HCl three times and then dried over $MgSO_4$ and reduced in vacuo. Silica gel column chromatography (hexane/EtOAc 2:1 + 1% (v/v) AcOH) then afforded 3.36 g (16.5 mmol, 50%) of the product as an off-white solid with identical ^{1}H NMR spectrum as reported in the literature.

<u>Compound 4</u>

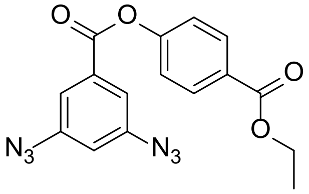

110 mg 3,5-diazidobenzoic acid (0.54 mmol, 1 equiv.), 90 mg ethyl paraben (0.54 mmol, 1 equiv.), 111 mg N,N′-dicyclohexylcarbodiimide (0.54 mmol, 1 equiv.) and 7 mg 4-dimethylaminopyridine (0.054 mmol, 0.1 equiv.) were dissolved in 10 mL anhydrous DCM and reacted under N_2 for 2 d. Afterwards, the solution was filtered, dried over $MgSO_4$, reduced in

vacuo and purified by silica gel column chromatography (hexane/Et_2O 85:15). This afforded 159.5 mg of product (0.47 mmol, 87%) as a white solid.

The ^{1}H NMR spectrum is consistent with literature in which the compound was obtained via a different synthetic route.[7]

Compound 5.XB

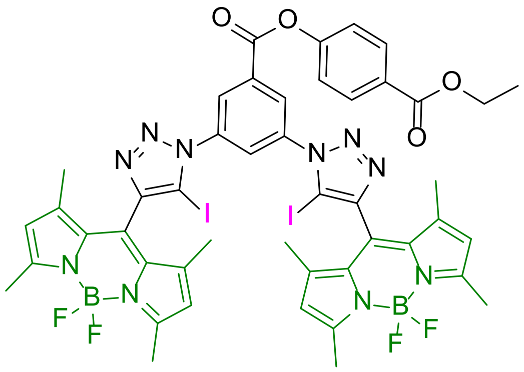

23 mg **4** (0.0655 mmol, 1 equiv.), 60 mg **3a** (0.151 mmol, 2.3 equiv.) 5 mg $Cu(CH_3CN)_4PF_6$ (0.0131 mmol, 0.2 equiv.) and 7 mg TBTA (0.0131 mmol, 0.2 equiv.) were dissolved in 5 mL anhydrous DCM and stirred overnight under N_2 in the dark. TLC indicated incomplete reaction, thus another 5 mg $Cu(CH_3CN)_4PF_6$ (0.0131 mmol, 0.2 equiv.) and 7 mg TBTA (0.0131 mmol, 0.2 equiv.) were added and the reaction stirred overnight. To this reaction mixture ~40 mL DCM were added and the solution was then washed with a half-concentrated solution of concentrated aq. NH_3 twice (~40 mL each), followed by washing with water twice (~40 mL each). The organic phase was dried over $MgSO_4$, reduced in vacuo and the product purified by preparative TLC (3% EtOAc in DCM) which afforded 74 mg (0.0645 mmol, 98%) of product as a red solid.

^{1}H NMR (400 MHz, $CDCl_3$) δ 8.70 (d, J = 2.0 Hz, 2H), 8.26 (t, J = 2.0 Hz, 1H), 8.23 – 8.15 (m, 2H), 7.42 – 7.30 (m, 2H), 6.07 (s, 4H), 4.41 (q, J = 7.1 Hz, 2H), 2.59 (s, 12H), 1.59 (s, 12H *partially overlapping with residual water*), 1.42 (t, J = 7.1 Hz, 3H).

^{13}C NMR (125 MHz, $CDCl_3$) δ 165.7, 161.9, 158.0, 153.8, 147.9, 142.2, 138.1, 132.7, 132.3, 131.6, 129.3, 128.3, 127.2, 126.5, 122.1, 121.6, 82.4, 61.5, 15.0, 14.5, 14.2.

HR-MS: (ESI+) $C_{46}H_{40}B_2F_3I_2N_{10}O_4$ $[M-F^-]^+$: calculated: 1129.1462. Found: 1129.1453.

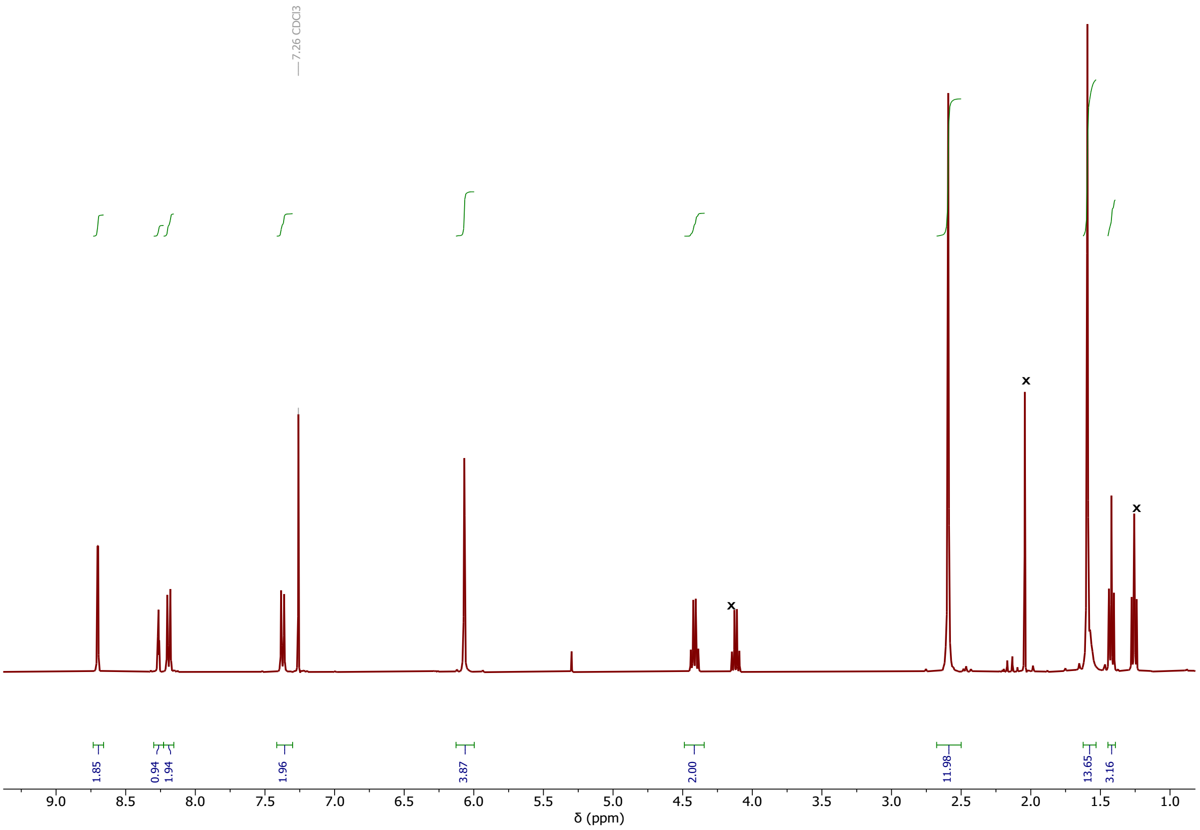

Figure S1. ^{1}H NMR spectrum of **5.XB** (400 MHz, $CDCl_3$). The peaks marked with an X arise from residual EtOAc.

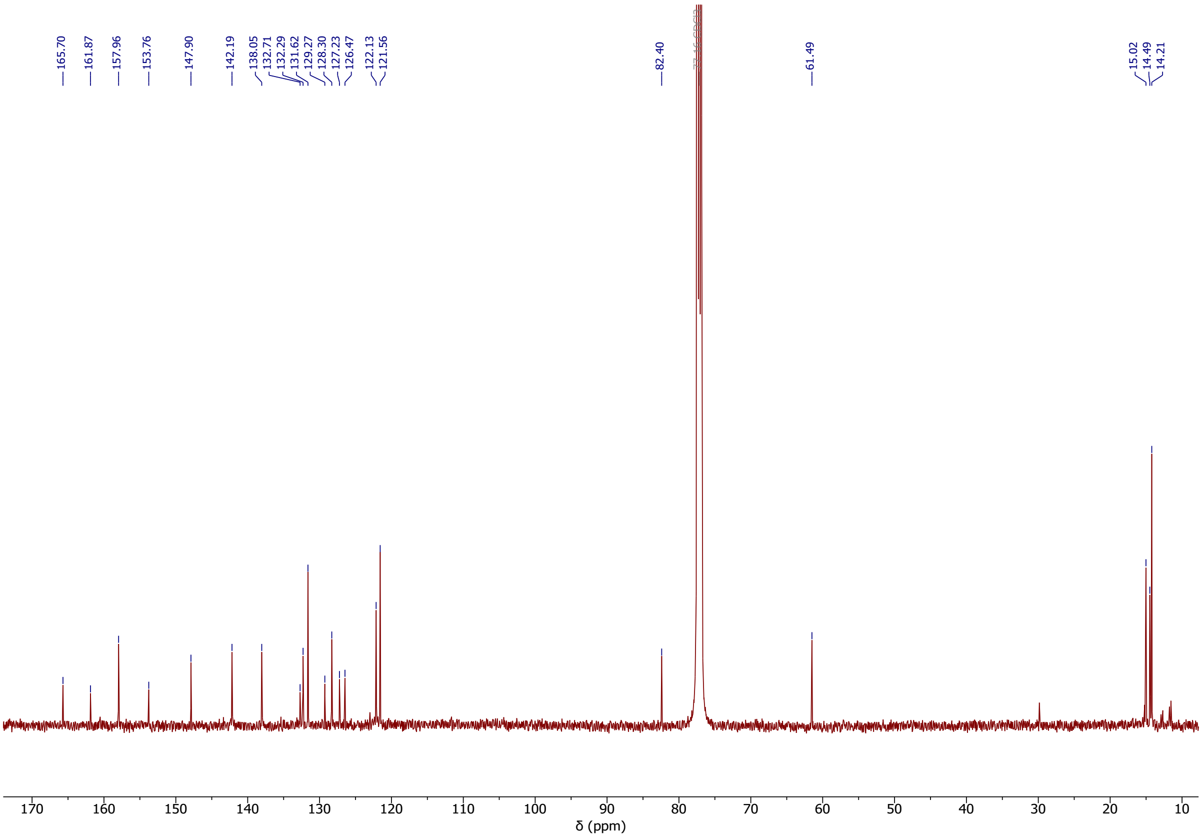

Figure S2. ^{13}C NMR spectrum of **5.XB** (125 MHz, $CDCl_3$).

Compound 5.HB

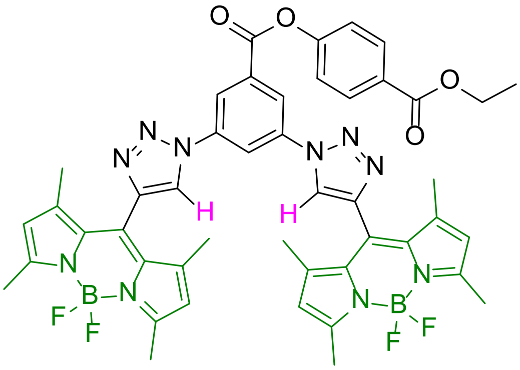

29 mg **4** (0.083 mmol, 1 equiv.), 52 mg **3b** (0.191 mmol, 2.3 equiv.) 6.2 mg $Cu(CH_3CN)_4PF_6$ (0.0166 mmol, 0.2 equiv.) and 8.8 mg TBTA (0.0166 mmol, 0.2 equiv.) were dissolved in 5 mL anhydrous DCM and stirred overnight under N_2 in the dark. To this reaction mixture ~50 mL DCM were added and the solution was then washed with a half-concentrated solution of NH_3 twice (~50 mL each), followed by washing with water twice (~50 mL each). The organic phase was dried over $MgSO_4$, reduced in vacuo and the product purified by preparative TLC (4% EtOAc in DCM) which afforded 70 mg (0.0775 mmol, 93%) of product as a red solid.

^{1}H NMR (400 MHz, $CDCl_3$) δ 8.85 (t, *J* = 2.1 Hz, 1H), 8.68 (d, *J* = 2.1 Hz, 2H), 8.31 (s, 2H), 8.24 – 8.13 (m, 2H), 7.37 – 7.32 (m, 2H), 6.05 (s, 4H), 4.41 (q, *J* = 7.1 Hz, 2H), 2.58 (s, 12H), 1.58 (s, 12H), 1.42 (t, *J* = 7.1 Hz, 3H).

^{13}C NMR (125 MHz, $CDCl_3$) δ 165.7, 162.2, 157.5, 153.8, 142.6, 142.3, 138.5, 133.5, 132.4, 131.6, 129.2, 127.0, 122.1, 121.6, 121.5, 121.0, 116.4, 61.5, 14.9, 14.7, 14.5.

HR-MS: (CI+) $C_{46}H_{42}B_2F_3N_{10}O_4$ $[M-F^-]^+$: calculated: 877.3529. Found: 877.3518.

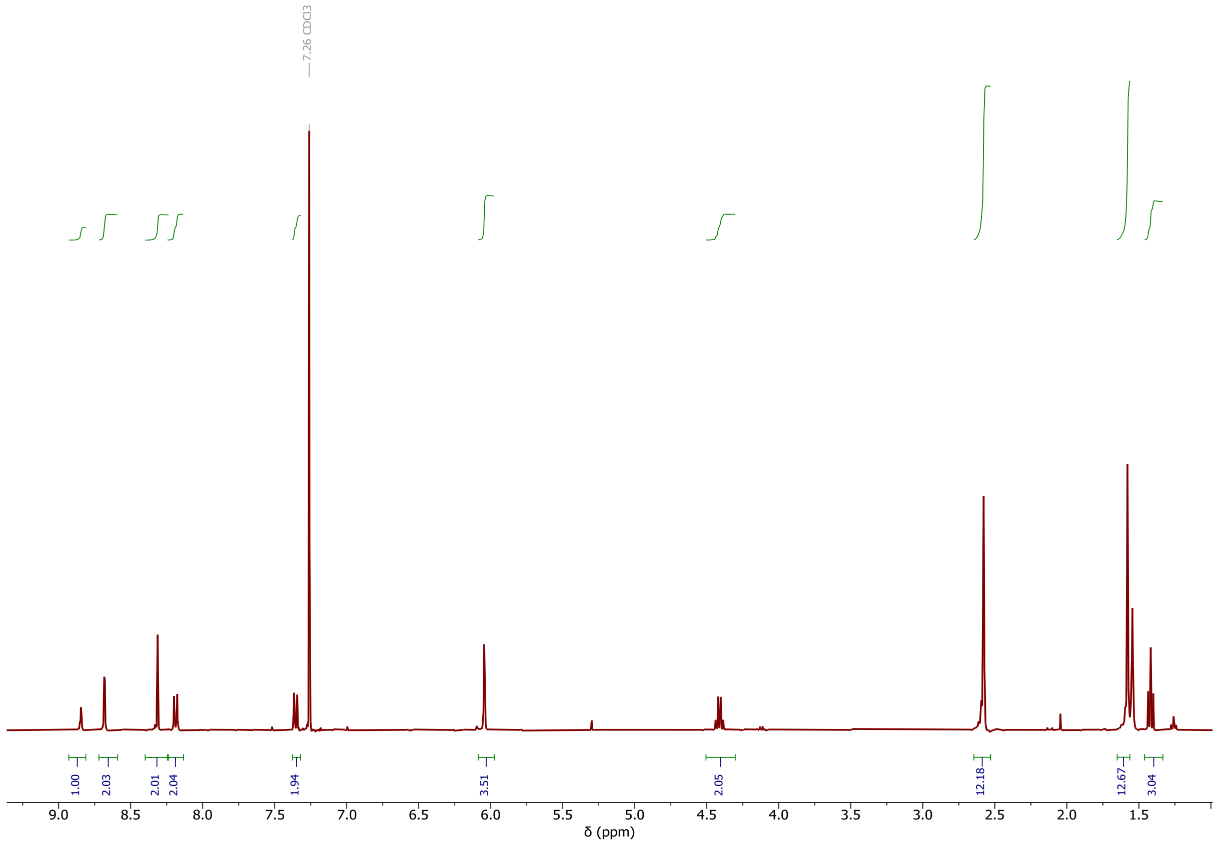

Figure S3. ^{1}H NMR spectrum of **5.HB** (400 MHz, $CDCl_3$).

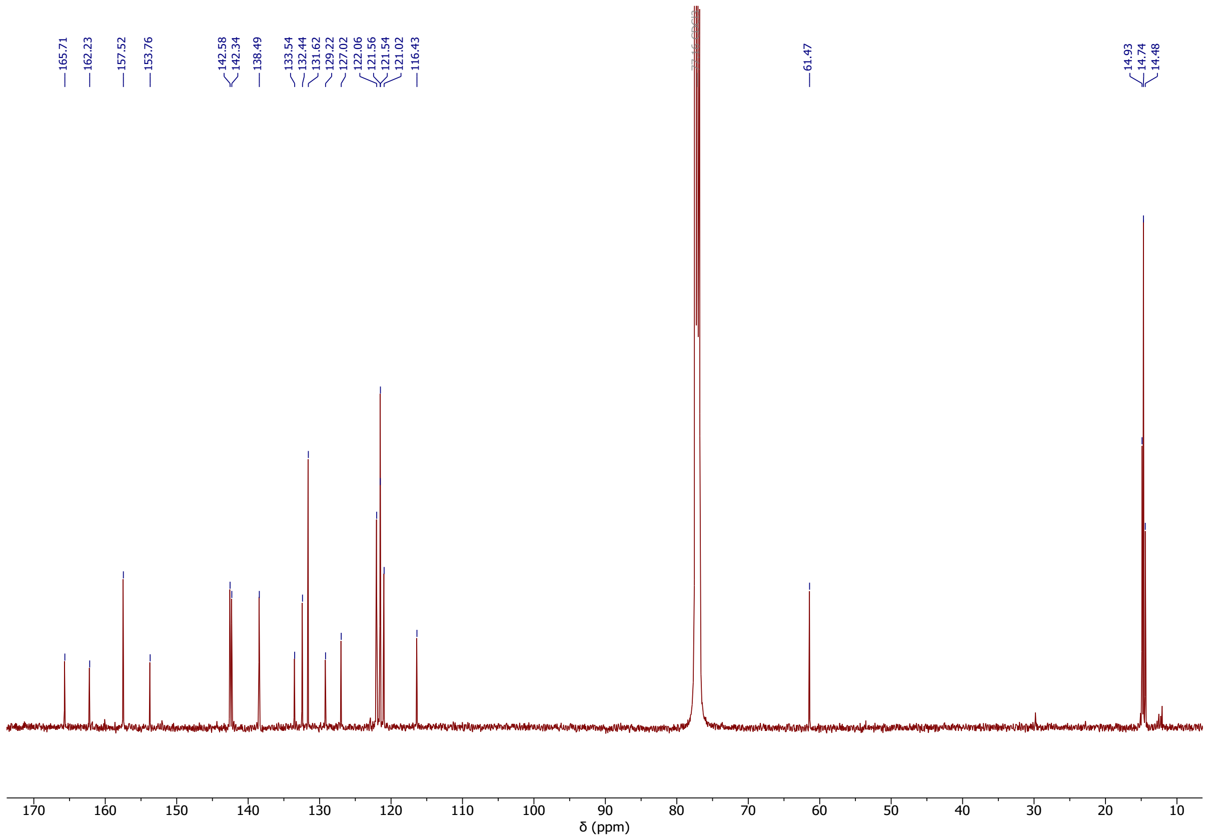

Figure S4. ^{13}C NMR spectrum of **5.HB** (125 MHz, $CDCl_3$).

<u>Compound 1.XB$_{PEG}$</u>

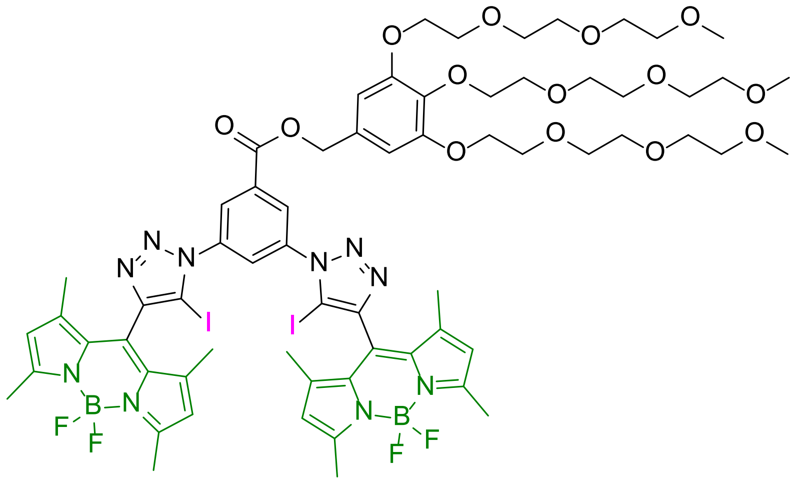

30 mg of **5.XB** (0.026 mmol, 1 equiv.) and 31 mg **6** (0.052 mmol, 2 equiv.) were dissolved in 4 mL anhydrous 1,2-dichloroethane. A few drops of triethylamine were then added and the reaction stirred at 50 °C under N_2 in the dark for 2 d. The solution was then partitioned between ~20 mL DCM and ~20 mL water and washed with water twice (~20 mL each), whereafter the organic phase was dried over $MgSO_4$ and reduced in vacuo. The product was purified by preparative TLC (5% MeOH in DCM) and isolated as a red solid (6.5 mg, 0.004 mmol, 16%).

^{1}H NMR (400 MHz, $CDCl_3$) δ 8.55 (d, *J* = 2.0 Hz, 2H), 8.19 (t, *J* = 2.0 Hz, 1H), 6.72 (s, 2H), 6.07 (s, 4H), 5.39 (s, 2H), 4.15 (m, 6H), 3.83 (t, *J* = 5.0 Hz, 4H), 3.78 (t, *J* = 5.1 Hz, 2H), 3.70 (m, 6H), 3.67 – 3.56 (m, 12H), 3.52 (m, 6H), 3.36 (s, 3H), 3.34 (s, 6H), 2.59 (s, 12H), 1.58 (s, 12H, *partially overlapping with residual water*).

^{13}C NMR (151 MHz, $CDCl_3$) δ 157.9, 153.1, 147.8, 142.3, 137.9, 133.6, 132.3, 127.9, 122.1, 108.8, 82.7, 72.6, 72.1 (2x), 71.0, 70.8 (2x), 70.7, 69.9, 69.3, 68.3, 59.2, 15.0, 14.2. *(Due to low sample amount and high MW not all aromatic peaks could be clearly resolved)*

HR-MS: (ESI+) $C_{65}H_{80}B_2F_4I_2N_{10}O_{14}Na$ $[M+Na^+]^+$: calculated: 1599.3965. Found: 1599.3924.

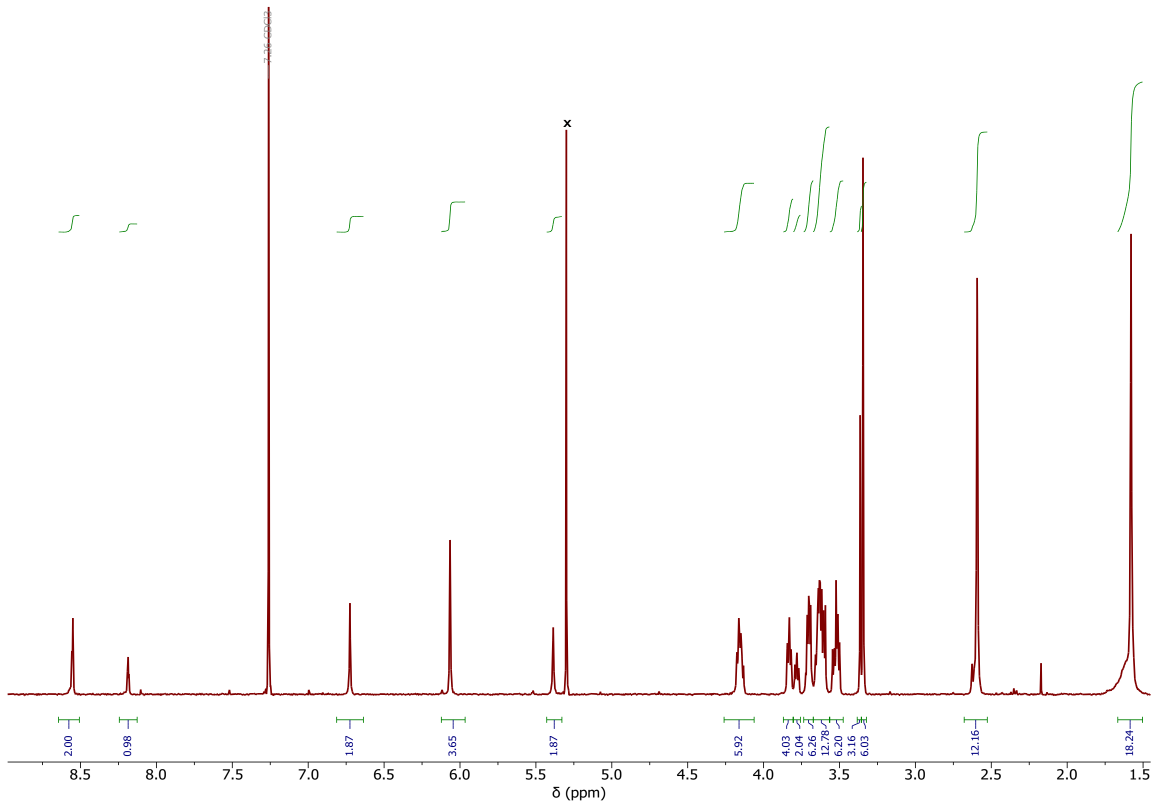

Figure S5. ^{1}H NMR spectrum of **1.XB$_{PEG}$** (400 MHz, $CDCl_3$). The peak marked with an X arises from residual DCM.

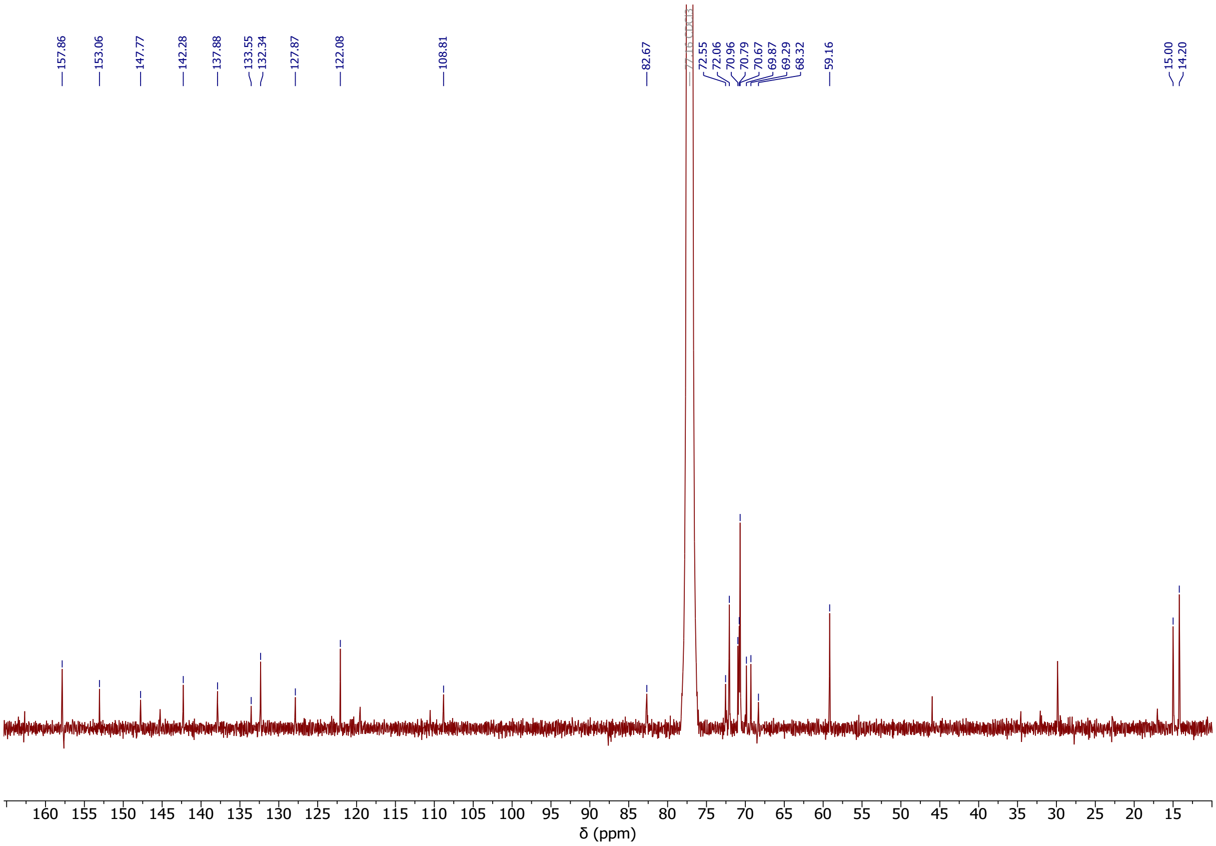

Figure S6. ^{13}C NMR spectrum of **1.XB$_{PEG}$** (125 MHz, $CDCl_3$).

Compound 1.HB$_{PEG}$

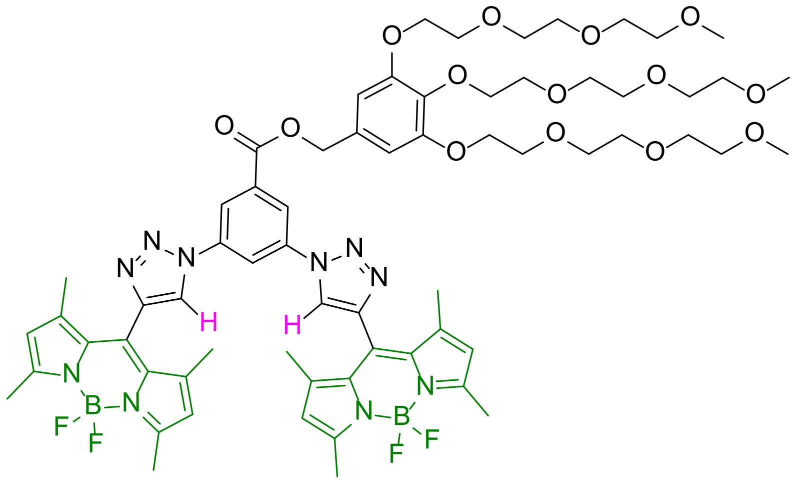

30 mg of **5.HB** (0.0335 mmol, 1 equiv.) and 40 mg **6** (0.067 mmol, 2 equiv.) were dissolved in 4 mL anhydrous 1,2-dichloroethane. 10 mg (14 µL) of triethylamine (0.1 mmol, 3 equiv.) were then added and the reaction stirred under N_2 in the dark overnight. TLC indicated only minor conversion, thus the reaction was heated to 50 °C for 2 d. The solution was then partitioned between ~25 mL DCM and ~25 mL water and washed with water twice (~25 mL each), whereafter the organic phase was dried over $MgSO_4$ and reduced in vacuo. The product was then purified by preparative TLC (5% MeOH in DCM) and isolated as a red solid (25 mg, 0.0189 mmol, 56%).

^{1}H NMR (400 MHz, $CDCl_3$) δ 8.78 (t, J = 2.1 Hz, 1H), 8.52 (d, J = 2.1 Hz, 2H), 8.32 (s, 2H), 6.73 (s, 2H), 6.03 (s, 4H), 5.35 (s, 2H), 4.16 (m, f6H), 3.83 (m, 4H), 3.78 (m, 2H), 3.74 – 3.67 (m, 6H), 3.67 – 3.58 (m, 12H), 3.56 – 3.47 (m, 6H), 3.36 (s, 3H), 3.34 (s, 6H), 2.57 (s, 12H), 1.56 (s, 12H).

^{13}C NMR (125 MHz, $CDCl_3$) δ 163.9, 157.4, 153.0, 142.7, 142.1, 138.2, 134.5, 132.5, 130.3, 127.3, 123.5, 122.0, 121.6, 120.7, 115.9, 109.3, 72.5, 72.1, 72.0, 70.9, 70.8 (2x), 70.7, 69.9, 69.2, 59.1, 14.9, 14.7.

MS: (ESI+) $C_{65}H_{82}B_2F_4N_{10}O_{14}Na$ [M+Na$^+$]$^+$: calculated: 1347.6046. Found: 1347.6000

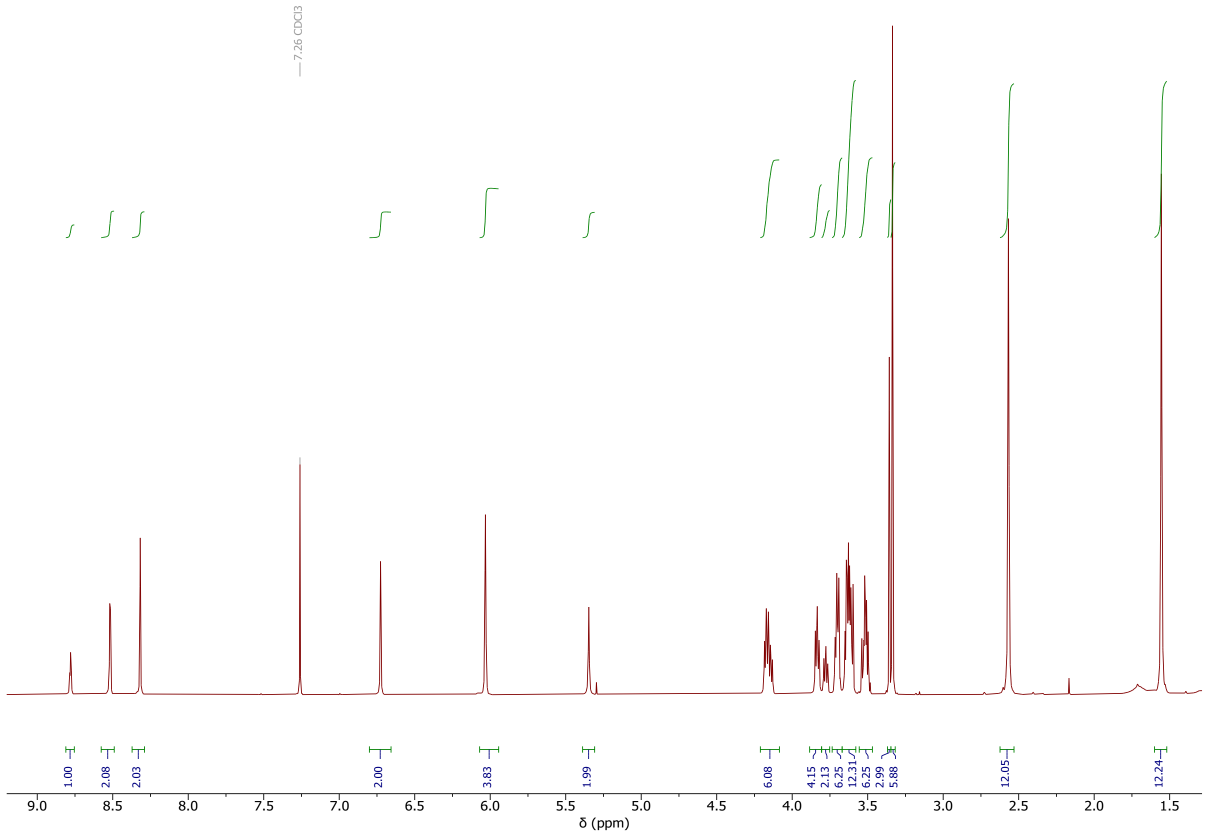

Figure S7. ^{1}H NMR spectrum of **1.HB$_{PEG}$** (400 MHz, $CDCl_3$).

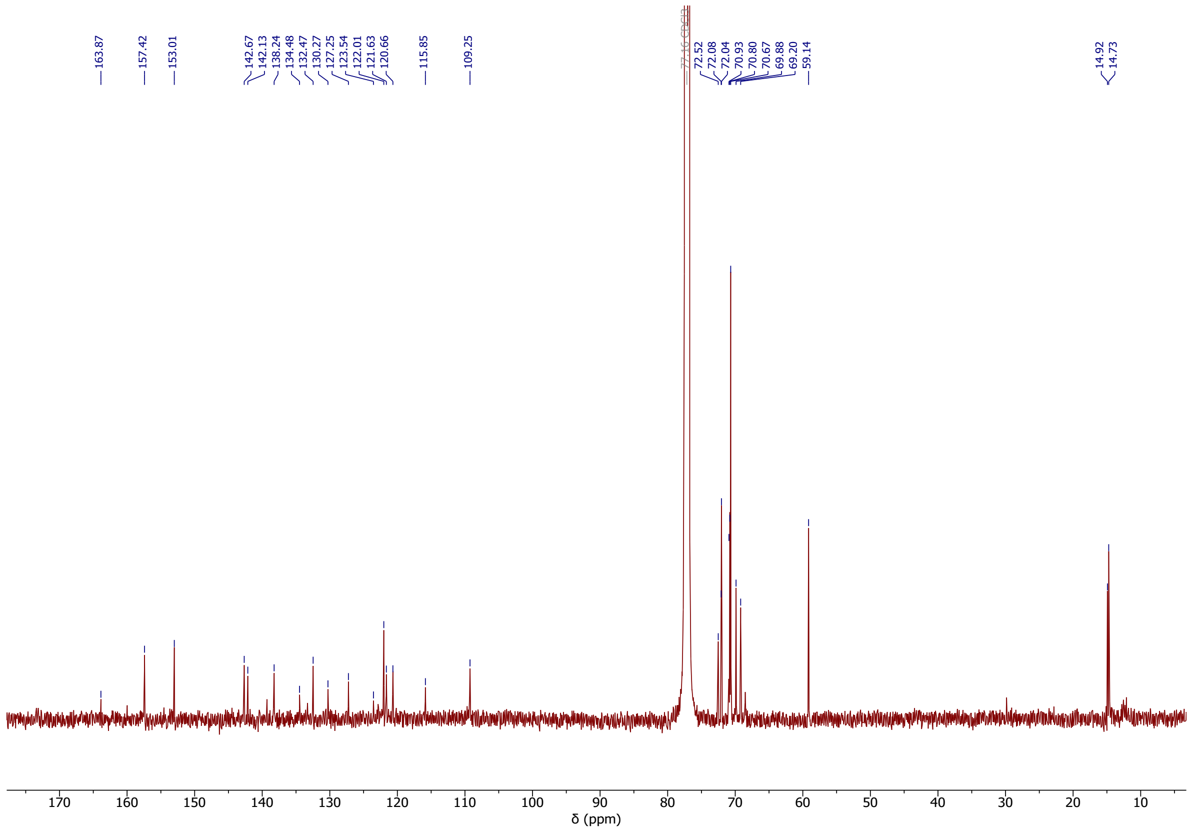

Figure S8. ^{13}C NMR spectrum of **1.HB$_{PEG}$** (125 MHz, $CDCl_3$).

3. Surface-Functionalisation

To enable highly reproducible formation of the receptive, fluorescent films, we utilized commercial amine-functionalized glass slides (“2D-Amino”, 25 x 75 x 1 mm) with an ultra-flat surface and low inherent fluorescence from PolyAn (Germany), containing a thin silane-based amine-film on both sides of the glass slide. Prior to modification, these slides were cut into smaller pieces (25 x ~9 x 1 mm) with a diamond cutter to fit into the holder/cuvette (see below) and then thoroughly washed with water, EtOH and DCM and then dried under a stream of nitrogen. The dried glass slides were then immersed in a 100 µM solution of the active-ester (**5.XB/HB**) in anhydrous DMF overnight in the dark. Subsequently, the glass substrates were rinsed with copious amounts of DCM, EtOH and water and stored in water at 4 °C in the dark until further use.

4. 3D-printed Holder for Optical Measurements

As briefly discussed in the main text, the 3D-printed holder was designed in a way to enable consistent fluorescence measurements of the functionalised glass slides in a stirred solution, to which a titrant solution could be added without disassembly or changing the position of the glass slide. This is important, because we found that even minor changes in the position of the slide significantly altered the fluorescence intensity.

The slide holder was designed in an iterative manner, whereby we adjusted various parameters to enable consistent and stable placement of the glass slides (25 x ~9 x 1 mm) in the cuvette. The final adapted design (Figures S9-10) consisted of two main sections: first a cubic body with dimensions of 9.5 x 9.5 x 15 mm ($\ell \times w \times h$) to fit into standard 10 x 10 mm square, open quartz cuvettes (inner diameter). The lower section of this body is equipped with a 5 mm deep slit with a width of 1.15 mm at a defined angle to squarely hold glass slides (1 mm thickness) without the need for applying too much pressure, while maintaining the slide tightly in place. Various angles were tested whereby 30° and 60° geometries generally gave best results in terms of minimizing artefacts from scattered light. In the most common iteration, a 30° “through” angle was chosen, whereby the excitation and emission beam were on opposite sides of the glass slides, i.e. the light had to first pass through the slide. This minimizes total reflection of the excitation light towards the detector. In principle the holder can be used either in air-filled or solvent-filled cuvettes, however, in our case we found that the quality of the fluorescence spectra was improved in the presence of solvent (less light scattering).

To stabilise the slide and holder in/on top of the cuvette, the upper section of the holder was designed with a downward-tapering square base with top surface dimensions of 12.31 x 12.31 mm ($\ell \times w$) that extends 10.1 mm to where it forms the base for the lower cubic body (9.5 x 9.5 mm; $\ell \times w$). To enable addition of solutions through the holder, two holes of 3 mm diameter were incorporated into the holder along the opposite diagonal of the slits and

passing straight through the whole length of the holder. Figures S9-10 display different views of the 3D model of the holder which was used for 3D printing, including all relevant distances.

The holders were designed using Autodesk Fusion360, then printed using a FormLabs Form 2 3D printer using FormLabs Tough 2000 resin, which is chemically resistant to a range of organic solvents. Notably, there can be slight variations between the dimensions of the design and the actual 3D printed holders (± 5%).

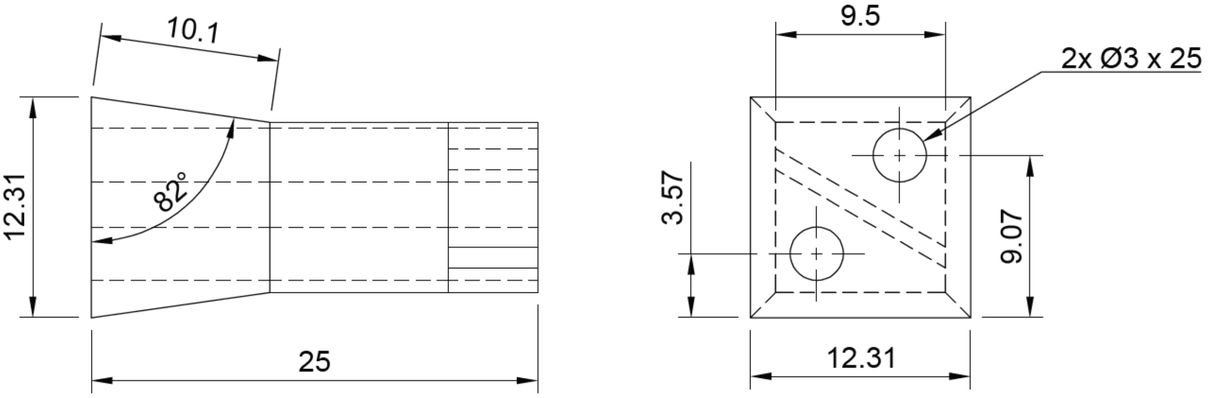

Figure S9. Model of the 3D-printed sample holder with relevant dimensions (all in mm). Left: side view. Right: view from bottom.

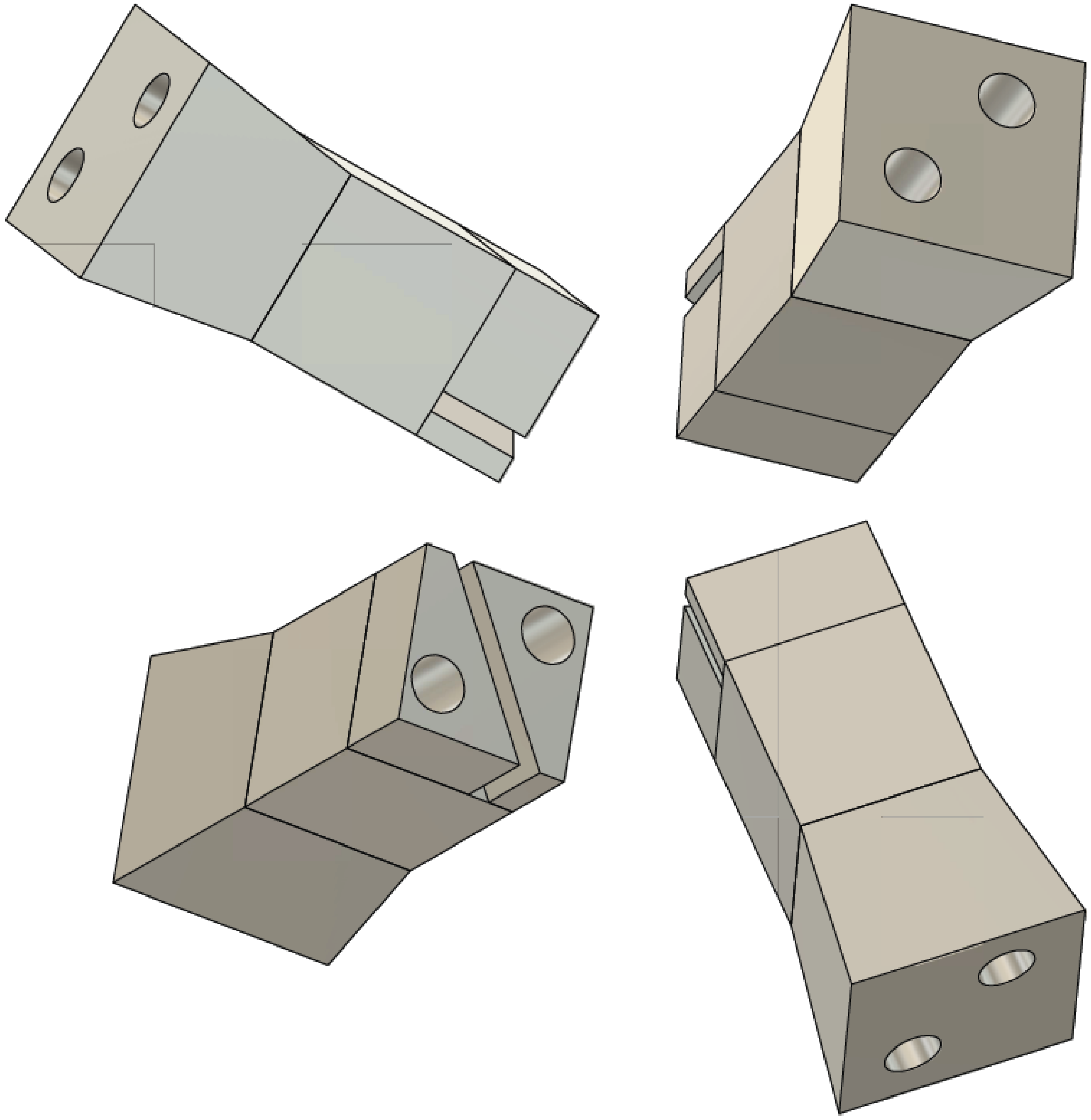

Figure S10. Model of the 3D-printed sample holder from different viewing angles.

5. Optical Measurements

Shown in Figures S11-12 are the optical properties of **1.XB/HB$_{PEG}$** in different solvent systems (pure water and water/CH_3CN mixtures). From both the absorbance and fluorescence profiles it is clear that above 70% water significant aggregation is observed which leads to broadened absorption and significantly decreased fluorescence. In pure water, addition of the detergent Triton X-100 induces break-up of the aggregates, which leads to a qualitative recovery of the optical properties observed in the dissolved state, i.e. in organic solvent. Interestingly, before strongly diminishing above 70% water, the fluorescence emission intensity of both **1.XB/HB$_{PEG}$** first increases upon addition of water (Figure S12).

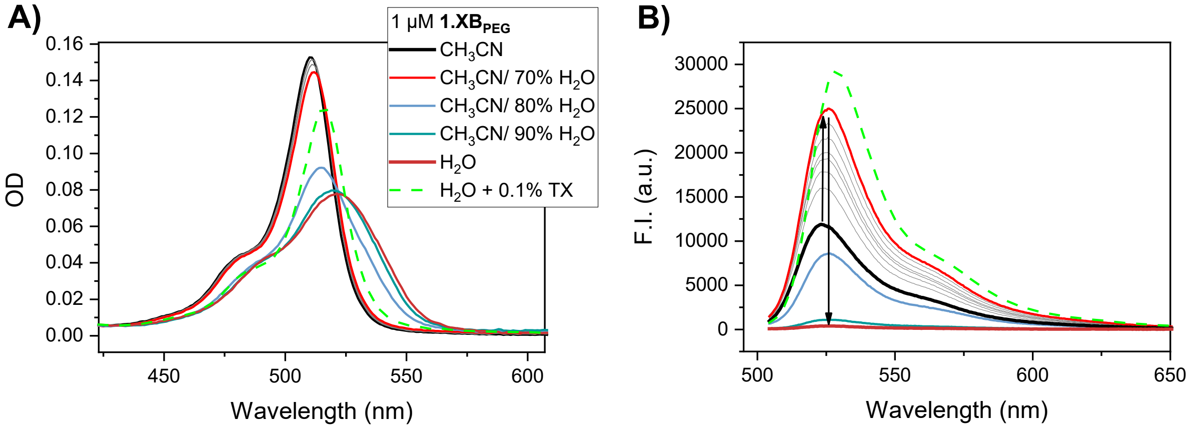

Figure S11. Optical properties of 1 µM **1.XB$_{PEG}$** in a range of solvent systems including CH_3CN, H_2O, CH_3CN/H_2O mixtures and H_2O + Triton X-100 (labelled "TX"). A) Absorbance. B) fluorescence emission intensity.

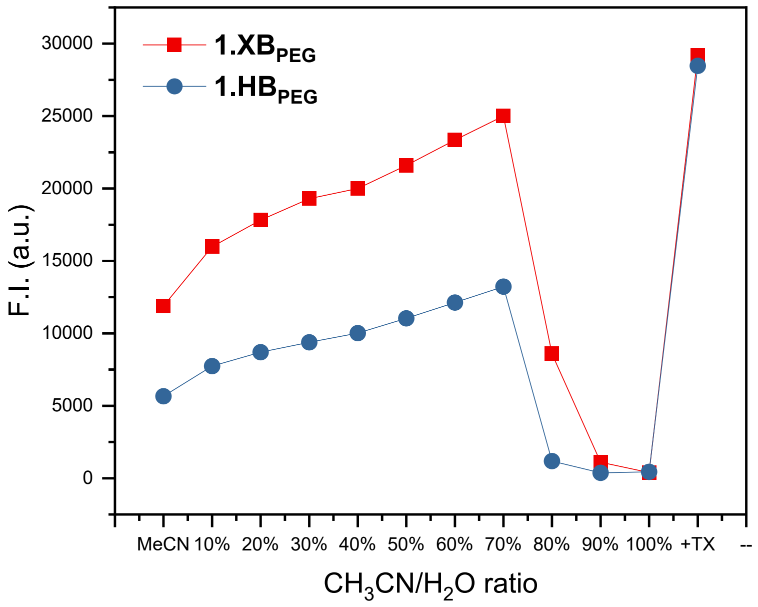

Figure S12. Fluorescence emission intensity at λ_{max} of 1 µM **1.XB/HB$_{PEG}$** in a range of solvent systems including CH_3CN, H_2O, CH_3CN/H_2O mixtures and H_2O + Triton X-100 (labeled as "TX"). Above 70% water the fluorescence intensity drops very significantly due to aggregation. In pure water, high "monomeric" fluorescence is only possible in the presence of a detergent such as Triton X-100.

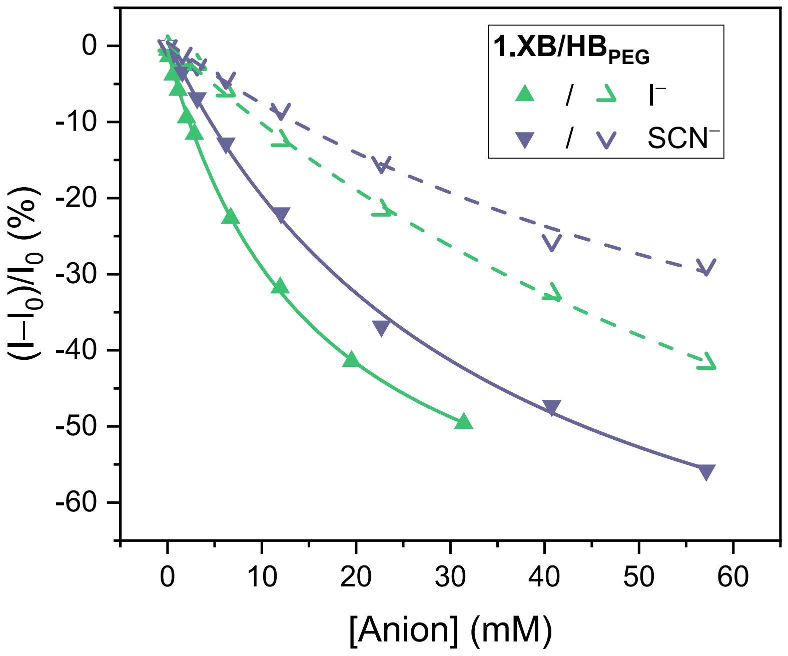

Figure S13. Relative fluorescence emission response of 1 µM **1.XB/HB$_{PEG}$** in H_2O/CH_3CN 7:3 upon addition of various anions. Filled symbols represent the XB receptor while empty symbols represent the HB system. The solid/dashed lines correspond to fits according to a 1:1 host-guest stoichiometric binding model.

Table S1. Anion binding constants K (M^{-1}) of **1.XB/HB$_{PEG}$** and **6.XB/HB** in acetone obtained by global fitting of the fluorescence binding isotherms to a 1:1 host-guest stoichiometric binding model.

	**1.XB$_{PEG}$**	**1.HB$_{PEG}$**	**6.XB**[2]	**6.HB**[2]
**Cl^-**	9980	200	7860	70
**Br^-**	12700	90	8600	28
**I^-**	8050	/	4550	/

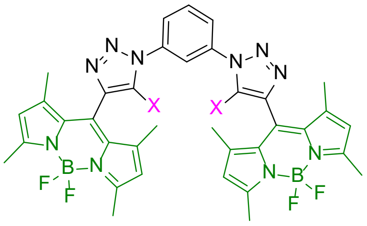

6.XB: X = I

6.HB: X = H

Figure S14. Chemical structures of **6.XB/HB**.

Table S2. Maximum relative response magnitude I_{max} for different sensors and anions obtained from fitting of the binding isotherms to either a 1:1 host-guest stoichiometric binding model (solution) or Langmuir isotherm (interface) at $[A^-] = \infty$.

	Acetone				Water			
	**1.XB$_{PEG}$**	**1.HB$_{PEG}$**	**2.XB$_{Glass}$**	**2.HB$_{Glass}$**	**1.XB$_{PEG}$[a]**	**1.HB$_{PEG}$[a]**	**2.XB$_{Glass}$**	**2.HB$_{Glass}$**
**Cl^-**	+100%	+306%	+98%	+70%	/	/	/	/
**Br^-**	+105%	+238%	+44%	~+50%[b]	/	/	/	/
**I^-**	+43%	/	/	/	−75%	−74%	−76%	−67%
**SCN^-**	/	/	/	/	−89%	−114%	−53%	−56%

a – for solubility reasons 30% CH_3CN were added for solution-phase anion binding studies. b – large fitting error/uncertainty

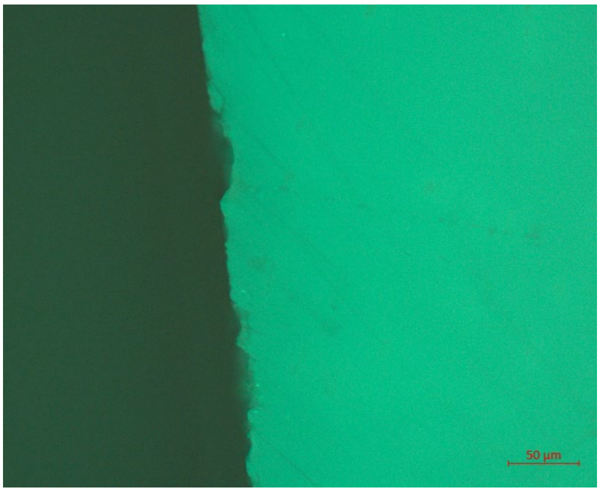

Figure S15. Fluorescent microscopy image of the edge of a **1.XB$_{Glass}$** glass slide (λ_{Exc} = 475 nm, ZEISS fluorescence microscope). The scale bar corresponds to 50 µm.

6. References

[1] P. Thordarson, *Chem. Soc. Rev.* **2011**, *40*, 1305-1323.

[2] A. J. Taylor, R. Hein, S. C. Patrick, J. J. Davis, P. D. Beer, *Angew. Chem. Int. Ed.* **2024**, *63*, e202315959.

[3] M. Albrecht, A. Lippach, M. P. Exner, J. Jerbi, M. Springborg, N. Budisa, G. Wenz, *Org. Biomol. Chem.* **2015**, *13*, 6728-6736.

[4] K. C. Elbert, D. Jishkariani, Y. Wu, J. D. Lee, B. Donnio, C. B. Murray, *Chem. Mater.* **2017**, *29*, 8737-8746.

[5] L.-H. Xie, M. P. Suh, *Chem. Eur. J.* **2013**, *19*, 11590-11597.

[6] A. T. Zill, S. C. Zimmerman, *Isr. J. Chem.* **2009**, *49*, 71-78.

[7] A. Borissov, I. Marques, J. Y. C. Lim, V. Félix, M. D. Smith, P. D. Beer, *J. Am. Chem. Soc.* **2019**, *141*, 4119-4129.
